# Microbial Contamination and Public Health: An Overview

**DOI:** 10.3390/ijerph19127441

**Published:** 2022-06-17

**Authors:** Alessia Tropea

**Affiliations:** Department of Research and Internationalization, University of Messina, Via Consolato del Mare, 41, 98100 Messina, Italy; atropea@unime.it

**Keywords:** microbiological contamination, food contamination, innovative technologies, public health, foodborne diseases, food hygiene, food safety, good hygienic, manufacturing practices

## Abstract

Food contamination with microbial agents can take place at any stage of the food chain, from farm to fork. For this reason, good hygienic and manufacturing practices must be followed along the entire food chain to prevent microbiological food contamination due to microbes which can cause high incidence of morbidity and mortality among consumers. Recent research have been focused on the implementation of innovative technologies for enhancing the quality and safety of food without compromising its organoleptic and nutritional characteristics. Studies should be addressed to the development of simple, less expensive, and fast tests for monitoring and controlling microbial food contamination, as well as to the development of new food manufacturing processes.

Currently, there has been an increasing interest around food hygiene and safety, as well as around the incidence of food borne diseases because of the strict correlation with Public Health. In this respect, microbiological food contamination with pathogens microorganisms, their persistence, replication and/or toxin production has become one of the major concerns to consumers, food industries and regulatory agencies all around the world. Food microbial contamination can occur at any stage of the food chain and could be avoided by applying good manufacturing practices, Hazard Analysis Critical Control Point (HACCP) notions, raw material control, and maintenance of the cold chain at the industry and retail levels [1,2]. It can be referred not just to the growth of undesired microbes but also to the production of toxic molecules resulting by their metabolism, such as mycotoxins [3], or biofilm formation [4].

Tightly associated with microbial contamination is food losses due to spoilage or waste [5]. As result of the human population global growing, the food demand is constantly increasing. The Food and Agriculture Organization of the United Nations carried out a study revealing that one-third (1.3 billion tons per year) of food production for human consumption is lost due to spoilage or waste [6]. The improper wastes management can represent a public health risk and cause environmental problems, such as diseases and air pollution [7]. Food-borne disease transmission has received more attention than food spoilage, due to the public heath resonance. However, food spoilage causes important economic losses, adverse food industry publicity, and diminution of human food supplies [1,8]. For these reasons, an appropriate food management is recognized as an essential prerequisite for sustainable development, also for the attainment of the global sustainability goals (SDGs 12 and 13) since it will allow for lower food waste generation, with a double advantage both from an economical and environmental point of view [9].

## 1. Food Born Diseases

Microbial food-borne disease (FDB) is one of the major concerns in terms of public health because of the high risk of microbial contamination of foods by several types of biological hazards, causing personal distress, preventable deaths, and avoidable economic burden [10,11]. Every year, at least two billion people worldwide are affected by FBD, for this reason these diseases are recognized among the greatest public health problems in the contemporary world [12]. According to the World Health Organization (WHO) [13] every year 23 million people in Europe become ill, and 5000 deaths are caused by FBD [14].

Every step, from farm to fork, can represent a possible risk point through the FBD may occurs (e.g., food production, processing, packaging, transportation, and consumption). Since consumers represent the endpoint of the food safety chain they have been referred to as the “final line of defense” in the prevention of FBD [15].

Among the most common symptoms caused by foodborne pathogens there are nausea, vomiting, abdominal pain/discomfort, diarrhea, fever, and lack of appetite [16]. It is possible control or avoid the food microbial contamination by the application of good manufacturing practices, a strict raw material control, and the maintenance of the cold chain at the industry and retail levels [17].

Several research have highlighted the need to carry out a food safety training and education for food handlers, because of the lack of knowledge on microbiological food hazards, food storage temperatures, cross contamination risks and the importance of personal hygiene. The implementation of the education process, practices and perspectives can contribute to the impact of good manipulation practices, guaranteeing the food safety and food quality to the consumer, reducing also the incidence of food borne disease [2,17].

## 2. Food Preservation Techniques

For enhancing the safety and increasing the shelf-life of food products several “traditional” food preservation techniques, such as chilling, freezing, nutrient restriction, reduction of water activity, acidification, pasteurization, sterilization, fermentation and chemical/biological antimicrobials have been used for controlling the microbial growth in foods [18,19]. Among these, thermal treatments are the most widely used methods for microbial inactivation, both in the food and beverage industries, for safety reasons. However, they adversely affect the organoleptic and nutritional properties of foods, often reducing the consumer acceptability [20]. The necessity of increasing the safety and quality of foods without compromising the nutritional, functional, and sensory food characteristics is today the major challenge that has addressed the use of new innovative technologies in food industries.

In this context, several innovative non thermal treatments/technologies, that do not need to increase the food products temperature for allowing bacterial inactivation, have been developed for enhancing the food safety and for increasing the shelf life of food products [21,22]. In this regard, Akbar et al. [23] reviewed the utilization of free or encapsulated natural antimicrobial compounds used singly or in combination with other technologies, in order to reduce the total microbial population of food products. In particular, the review is focused on loaded-nisin nanoparticles used in order to improve the food safety. Nano-carriers containing nisin, are referred to nano-liposomes, nano-emulsions, solid lipid nanoparticles, lipid nano-carriers, bio-polymeric nano-particles, and nano-fibers, that can be applied as a suitable antimicrobial matter in food formulations and in the food packaging materials. Several advantages can be obtaining by the application of this technique, for example sustained release, preventing undesirable interactions, and providing a high stability and efficient antimicrobial activity during food storage, preserving sensory properties, nutritional, and functional characteristics of food products [24].

Other incoming technologies for increasing the safety and shelf-life of food are used by the food industry. An example is represented by the utilization of High Hydrostatic Pressure (HHP), a non-thermal technology able to inactivate a wide range of food pathogens microbes and harmful food enzymes, by applying pressures above 100 MPa to the product through mechanically pressurized water, resulting in a minimal impact on the overall quality, nutritional properties, and food flavors. It has been previously reported that the utilization of pressure ranging between 300–600 MPa can efficiently prevent the microorganisms’ growth, guaranteeing the overall quality of food products [25].

Currently, a novel technology for microbial inactivation is becoming more popular among the scientific community: antimicrobial photodynamic treatment (aPDT). The action mechanism for microbial inactivation of aPDT is ascribable to the utilization of visible or near-infrared light with a photosensitizer (PS) in the presence of molecular oxygen for generating reactive oxygen species (ROS). The formation of ROS is the direct responsible of the cell death. Since reactive oxygen species can react with a several biomolecules and cell structures, it is possible the emergence of resistance to aPDT that remains to be under control, and for this reason further studies are required. Moreover, the utilization of non-toxic PS and harmless visible light makes aPDT a promising alternative for food applications. The aPDT shows an important range of application, in fact it is able to inactivate all types of microorganisms, including both Gram-positive and Gram-negative bacteria (vegetative cells and spores), yeasts, molds, protozoa, and viruses [1].

Woo-Ju et al. [26] report a study regarding the non-thermal effects of microwave radiation, pointing out that microwave sterilization is able to reduce the presence of pathogens in foods and to inactivate the enzymes, preserving at the same time the nutritional properties of foods [27]. Several factors can influence the microwave efficiency on microbial inactivation, such as the applied power, the electric field frequency, the time of the treatment, as well as the geometry and the dielectric properties of the product. Today, the number of studies carried out on this topic is still limited, for this reason it is important address new research on the optimization of treatment conditions for enhancing the utilization of microwave technology for reducing the microbial food contamination [24].

In addition, food irradiation has received much attention as non-thermal technology, and it can be defined as the application of ionizing radiation in small doses suitable as a decontaminant technology for improving the food safety and increasing the shelf-life [28] through the bacterial inactivation caused by their DNA damaging. However, due to the possibility of radioactivity generation in foods [29] the utilization of this technique results controversial. Moreover, this technique shows important drawbacks, due to the low penetrating power of irradiation for controlling pathogenic bacteria, resulting in a limited efficiency, especially when used on food having irregular surfaces. Therefore, it has been suggested that the utilization of irradiation, in combination with other technologies and methods, can represent a suitable practice to be adopted against microbial contamination in foods [24].

Currently, cold-plasma technology in the food sector is deeply recommended for inactivating harmful microorganisms potentially presented in foods including spore forming and spoilage microbes/pathogens. [25,30]. Several studies shown that cold-plasma, also defined non-thermal plasma (NTP), is able to destroy microorganisms on several kinds of food, packaging materials, and process equipment under atmospheric conditions [30,31,32,33,34]. The NTP is able to inactivate the microorganisms through the oxidation stress or the physical lysis caused by reactive species, determining the leakage and/or the release of the intracellular material [31] or resulting in genomic DNA damage [32]. The voltage, frequency, mode of exposure, gas mixture, treatment time, electrode type, as well as the microorganism cell membrane characteristics, the type of product and the bacterial location are all factors that need to be considered for the evaluation of the anti-microbial efficiency of NTP [33,34].

Another attracting technology for controlling the bacterial contamination of the surfaces in contact with foods, for decreasing the cross-contamination, is represented by the ozone utilization. Ozone is currently defined as a promising alternative to chlorine substances, since no residual components can be detected on foods after ozone treatment [35]. Ozone can be used against Gram positive and Gram negative bacteria as well as yeast and molds, and it has been also reported to be a virucidal agent. The action mechanism is due to the ability of ozone to oxidize the membrane of bacteria destroying them [25]. Strictly connected to the effectiveness of ozone treatment against microorganisms in food systems are several environmental factors such as temperature, pH, humidity rate, presence of additives and the amount of organic substances that can be detected around the cells [36]. One of the most important advantages of ozone treatment for food processing is that it can be rapidly decomposed into harmless molecular oxygen [37].

These are just few examples of innovative technologies and strategies that can be suitable for food microbial contamination control. In this scenario it is important highlight the necessity to implement new technologies and their application for fast and direct detection of the presence of microbial contamination and, of course, for preventing and/or reducing food microbiological contamination in all the food chain steps, enhancing food safety and quality. Moreover, a particular focus is request on the continuous educational process and practices for contributing to the effect of good manipulation procedures and guarantee the food safety, along all the food chain, and the food quality to the final consumers.
